# Intermittent energy restriction induces changes in breast gene expression and systemic metabolism

**DOI:** 10.1186/s13058-016-0714-4

**Published:** 2016-05-28

**Authors:** Michelle N. Harvie, Andrew H. Sims, Mary Pegington, Katherine Spence, Adam Mitchell, Andrew A. Vaughan, J. William Allwood, Yun Xu, Nicolas J. W. Rattray, Royston Goodacre, D. Gareth R. Evans, Ellen Mitchell, Debbie McMullen, Robert B. Clarke, Anthony Howell

**Affiliations:** Genesis Breast Cancer Prevention Centre, University Hospital of South Manchester NHS Foundation Trust, Southmoor Road, Manchester, M23 9LT UK; Applied Bioinformatics of Cancer, University of Edinburgh, Cancer Research UK Centre, Institute of Genetics and Molecular Medicine, Carrington Crescent, Edinburgh, EH4 2XR UK; Breast Cancer Now Research Unit, Institute of Cancer Sciences, Academic Health Science Centre, University of Manchester, Wilmslow Road, Manchester, M20 4BX UK; Manchester Institute of Biotechnology, School of Chemistry, University of Manchester, Princess St, Manchester, M1 7DN UK; The Christie NHS Foundation Trust, University of Manchester, Wilmslow Road, Manchester, M20 4BX UK

**Keywords:** Energy restriction, Gene expression, Metabolomics

## Abstract

**Background:**

Observational studies suggest weight loss and energy restriction reduce breast cancer risk. Intermittent energy restriction (IER) reduces weight to the same extent as, or more than equivalent continuous energy restriction (CER) but the effects of IER on normal breast tissue and systemic metabolism as indicators of breast cancer risk are unknown.

**Methods:**

We assessed the effect of IER (two days of 65 % energy restriction per week) for one menstrual cycle on breast tissue gene expression using Affymetrix GeneChips, adipocyte size by morphometry, and systemic metabolism (insulin resistance, lipids, serum and urine metabolites, lymphocyte gene expression) in 23 overweight premenopausal women at high risk of breast cancer. Unsupervised and supervised analyses of matched pre and post IER biopsies in 20 subjects were performed, whilst liquid and gas chromatography mass spectrometry assessed corresponding changes in serum and urine metabolites in all subjects after the two restricted and five unrestricted days of the IER.

**Results:**

Women lost 4.8 % (±2.0 %) of body weight and 8.0 % (±5.0 %) of total body fat. Insulin resistance (homeostatic model assessment (HOMA)) reduced by 29.8 % (±17.8 %) on the restricted days and by 11 % (±34 %) on the unrestricted days of the IER. Five hundred and twenty-seven metabolites significantly increased or decreased during the two restricted days of IER. Ninety-one percent of these returned to baseline after 5 days of normal eating. Eleven subjects (55 %) displayed reductions in energy restriction-associated metabolic gene pathways including lipid synthesis, gluconeogenesis and glycogen synthesis. Some of these women also had increases in genes associated with breast epithelial cell differentiation (secretoglobulins, milk proteins and mucins) and decreased collagen synthesis (TNMD, PCOLCE2, TIMP4). There was no appreciable effect of IER on breast gene expression in the other nine subjects. These groups did not differ in the degree of changes in weight, total body fat, fat cell size or serum or urine metabolomic markers. Corresponding gene changes were not seen in peripheral blood lymphocytes.

**Conclusion:**

The transcriptional response to IER is variable in breast tissue, which was not reflected in the systemic response, which occurred in all subjects. The mechanisms of breast responsiveness/non-responsiveness require further investigation.

**Trial registration:**

ISRCTN77916487 31/07/2012.

**Electronic supplementary material:**

The online version of this article (doi:10.1186/s13058-016-0714-4) contains supplementary material, which is available to authorized users.

## Background

Observational studies in women indicate that weight gain increases breast cancer risk and weight reduction decreases breast cancer risk [1–4]. In the Iowa Women’s Health Study, ≥5 % maintained weight loss resulted in 20–40 % reduction in postmenopausal breast cancer risk compared with women who continued to gain weight [1]. Multiple studies in rodent models also indicate that energy restriction reduces the risk of breast cancer [5]. This effect appears to be mediated by changes in a number of systemic factors and also local factors within breast cells. For example, systemically, energy restriction is associated with reductions in insulin, leptin and inflammatory markers. Within the breast, in general there is downregulation of metabolic pathways associated with anabolism and upregulation of pathways associated with catabolism [6–8]. The aforementioned data suggest energy restriction would be useful for breast cancer prevention in overweight and obese women. However, poor adherence to continuous energy restriction (CER) means energy restriction is difficult to implement.

We previously reported that daily 60 % (3350 kJ) CER for one menstrual cycle (four to five weeks) reduced weight, biomarkers of breast cancer risk and altered gene expression in breast and abdominal adipose tissue [9]. Daily 60 % energy restriction is not a viable strategy for risk reduction in the general population as it is difficult to sustain. We demonstrated that intermittent energy restriction (IER), which involves strict 65 % energy restriction for two days and normal healthy eating with a Mediterranean diet for five days each week is achievable and is associated with reduction in weight, body fat [10] and insulin resistance [10, 11] amongst overweight/obese women. IER is an increasingly popular approach for weight loss but little is known about its systemic effects on metabolism or on the breast itself [12]. In the study reported here we examined the effects of IER within the breast by gene expression analyses and assessment of breast adipocyte size. Systemic effects were assessed by measuring insulin, lipids and serum and urinary metabolites. We also assessed the effects of IER on gene expression in lymphocytes to determine whether this could be used as an accessible surrogate of changes in breast gene expression. The gene expression and metabolomics findings with IER were compared to a previous study of daily 60 % CER for one menstrual cycle at our centre [9].

In this study, IER for two days per week resulted in marked weight loss over four to five weeks and in multiple systemic metabolic alterations which changed most at the end of the two restricted days and predominately returned towards baseline at the end of the five unrestricted days of IER. Gene expression analysis of breast tissue identified two distinct groups of participants; 11 out of 20 women displayed changes involving downregulation of epithelial mRNAs associated with aspects of breast metabolism, whereas the remaining 9 women did not have these consistent changes. Gene expression changes in breast tissue did not correlate with those in lymphocytes. Changes in breast gene expression with four to five weeks of IER (two days of 65 % energy restriction per week, which achieved an overall 45 % energy restriction) appeared to vary between women and were not as consistent as changes observed previously after daily 60 % CER for one menstrual cycle [9].

## Methods

### Subjects

Twenty-four premenopausal overweight or obese women (body mass index (BMI) 24–34 kg/m^2^ and body fat percentage 30–42 %), aged 35–45 years, and at increased risk of breast cancer (greater than one in six lifetime risk) were recruited from the Genesis Family History Clinic at the University Hospital of South Manchester, UK. In addition to the above, entry criteria included a normal mammogram within 12 months, estimated visual assessed breast density ≥30 % to ensure epithelium was obtained in the core biopsy, stable or increasing weight, and sedentary lifestyle (participating in ≤40 minutes of moderate activity per week) and regular menstrual cycles. Exclusion criteria included; restrictive, or high phytoestrogen-supplemented diets, previous use of tamoxifen, regular use of anti-inflammatory, anticoagulant, anti-platelet or oral contraceptive medication, pregnancy or planning a pregnancy, hysterectomy, co-morbid conditions such as a previous diagnosis of cancer, diabetes, ischaemic vascular disease, thyroid disease or psychiatric disorders that would limit adherence to the dietary programme and factors that may affect lymphocyte function, i.e. underlying autoimmune, inflammatory or allergy conditions, inoculations or blood donation within the past two months.

The study was reviewed by the North West 10 Research Ethics Committee, Greater Manchester North (09/H1006/33). All participants provided written informed consent prior to participation. The trial registration number is ISRCTN77916487 (www.isrctn.com/ISRCTN77916487).

### Intermittent energy restriction for one menstrual cycle

The IER intervention was designed to produce an overall 25 % energy restriction below estimated requirements (calculated from estimated resting energy expenditure multiplied by a physical activity level of 1.4) [13, 14] and ranged from 5984 to 7560 kJ/day for the participants. The IER included two consecutive days of 65 % energy restriction (approximately 2700 kJ, 100 g carbohydrate and 50 g of protein per day) and a Mediterranean-type diet that met subjects’ estimated energy requirements for the remaining five days of the week (range 7300–9500 kJ/day). Each energy-restricted day included four 80-g portions of vegetables and one 80-g portion of fruit, and six portions of low-fat dairy produce, e.g. 2 pints of semi-skimmed milk or equivalent low-fat yoghurt or cottage cheese. On the five subsequent days the women were asked to consume a Mediterranean style diet, which provided 45 % of energy from low-glycaemi-index carbohydrates, 30 % from fat (15 % monounsaturated fat, 7 % from saturated fat, 8 % from polyunsaturated fat) and 25 % energy from protein as described previously [10, 11]. Women commenced the diet within two days of their baseline assessment and followed the diet for one menstrual cycle (four to five weeks). Timing of trial assessments was standardised to one phase of the menstrual cycle. Of the 24 women who entered the study, 23 completed the energy restriction for one menstrual cycle. Nineteen of these started and finished in the follicular phase of their cycle, and four started and finished in the early luteal phase. Diets were not provided to participants but were self-selected, guided by detailed individualised food portion lists, meal plans and recipes. Participants were asked to keep daily food diaries throughout the study period and were monitored weekly by the study dietitians to maximize compliance. Participants were asked to maintain their sedentary lifestyle.

### Trial assessments

**Fig. 1:**
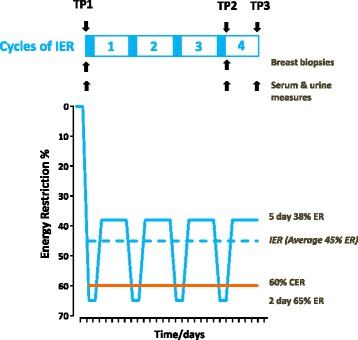
Degree of dietary energy restriction during four to five weeks of intermittent energy restriction (*IER*) and continuous energy restriction (*CER*). The IER cohort undertook 65 % energy restriction (*ER*) on two consecutive days per week and additionally restricted their energy intake to an average of 38 % below their baseline intake on the remaining five days of the week, which was unplanned (*blue solid line*). The planned and unplanned energy restriction resulted in an overall 45 % energy restriction over the one-month trial period (*blue dashed line*). For comparison our previous study of CER involved a 60 % daily energy restriction over a one-month period. *TP1* time point 1 (baseline): breast gene expression, adipocyte size, serum hormones, urine and serum metabolomics, weight and anthropometry. *TP2* time point 2 (immediately after two restricted days): breast gene expression, adipocyte size, serum hormones, urine and serum metabolomics, weight and anthropometry. *TP3* time point 3 (after 5 days of normal healthy eating): serum hormones, urine and serum metabolomics, weight and anthropometry

Weight and anthropometric measurements, breast biopsy (breast gene expression, breast adipocyte size), blood and urine samples (serum hormones, lipids and serum and urine metabolic markers) were conducted after an overnight fast at baseline before starting the diet (time point 1 (TP1)) and after four to five weeks of the diet on the morning immediately after the two day 65 % dietary restriction (time point 2 (TP2)). Weight, anthropometrics, blood and urine samples were also repeated at the end of the final week after five further unrestricted days (time point 3 (TP3)) (Fig. 1).

### Body measurements

Body weight and total body fat, percentage body fat, fat free mass (FFM) (bioelectrical impedance, Tanita, UK TBF180) and waist, hip and bust measurements were assessed using standard methods [11]. Dietary adherence was assessed throughout the four to five week study period with weekly food diaries. Physical activity was assessed using the International Physical Activity Questionnaire (IPAQ) [15] to confirm participants had remained sedentary throughout the study.

### Serum assays and metabolomics

Fasting insulin, glucose, low-dendity lipoprotein (LDL), high-density lipoprotein (HD)L, cholesterol and triglycerides were analysed in the Biochemistry Department at the University Hospital of South Manchester as described previously [10]. Fasting insulin and glucose were combined to calculate the insulin sensitivity index using the homeostasis model assessment (HOMA) [16]. Serum and plasma samples were aliquoted, stored at −80 °C, and batched so that all samples from a participant were included in the same assay. Serum estradiol and progesterone were assessed using electro-chemiluminescence (Abbott diagnostics, Abbott Ireland). Serum and urine samples were collected for metabolomics analysis using standard methods and respectively analysed at the Manchester Institute of Biotechnology, University of Manchester using four modes [17, 18]; liquid chromatography mass spectrometry (LCMS)-positive mode (LCMS+), LCMS-negative mode (LCMS–), gas chromatography mass spectrometry (GCMS) serum and GCMS urine.

### Breast biopsy procedure

Breast biopsies within dense areas of the breast were performed under radiographic guidance with the breast immobilised under the compression device. After infiltration of 2 % lidocaine, a small incision was made in the skin at the biopsy site, through which a 14-gauge biopsy needle was inserted to a depth estimated by the operator. Between seven and nine biopsy samples were obtained through the same skin incision and the direction of the needle was similar for each sample. The TP1 biopsies were taken from either the left or right breast side, which was chosen independently by computer randomisatio,n and the final TP2 biopsies were taken from the opposite side to eliminate potential gene expression changes related to the healing process. One half of two separate breast cores was fixed in 4 % formalin and embedded in paraffin blocks, and the remaining tissue was immediately snap frozen in liquid nitrogen and stored at –80 °C.

### Collection and analysis of lymphocytes

Blood samples from EDTA tubes were layered on top of Lymphoprep (Axis Shield Ltd, UK) and spun at 800 g for 20 minutes at 4 °C. Lymphocytes were then removed from the middle of the tube and spun once more at 1000 g for 10 minutes. RNA was extracted from the pelleted lymphocytes using Qiashredder columns and the RNAeasy Plus kit (Qiagen Ltd, UK) according to manufacturer’s instructions [19].

### Gene expression analysis

RNA was extracted from the breast samples with Qiazol by grinding breast tissue to a fine powder under liquid nitrogen and then chloroform and RNeasy columns (Qiagen Ltd, UK) as described previously [9]. Amplification of total RNA from breast tissue and lymphocyte samples was performed using the Nugen WT-Ovation™ Pico System RNA amplification kit (Nugen Technologies, Inc, Netherlands), labelled and hybridized to Affymetrix U133 plus2 GeneChips using manufacturers’ protocols. Gene expression data were analysed using packages within Bioconductor [20] implemented in the R statistical programming language. The gene expression data were summarised from CEL files using the original Affymetrix annotation or Ensembl gene identifiers using an alternative Chip Definition File (aCDF) [21] and normalised using Robust Multi-array Average algorithm [22] within the ‘affy’ package. Paired and unpaired rank products analysis [23] or significance analysis of microarrays (SAM) [24] methods using the siggenes package were used to identify differentially expressed genes with a 5 % false discovery rate (FDR). Cross-validation and the misclassification rate were assessed using prediction analysis of microarrays (PAM), implemented using the pamr package [25]. Kyoto Encyclopedia of Genes and Genomes (KEGG) pathways and Gene Ontology terms associated with the gene lists generated were explored using the Database for Annotation, Visualization and Integrated Discovery (DAVID) bioinformatics tool [26]. All raw and processed gene expression files are available from NCBI Gene Expression Omnibus (GEO) [GSE66161 and GSE66159].

### Assessment of breast biopsy composition

Breast biopsy composition was assessed to inform whether pre and post intervention samples had comparable composition to allow a comparison of gene expression. Three-micrometre sections were cut from the formalin-fixed paraffin-embedded breast biopsy cores, and stained with haematoxylin and eosin using a standard protocol. The proportions of fat, stroma and epithelium in each sample were estimated using Definiens Image Miner version 2.1.1 (Definiens AG, Germany) and refined using a training set of 12 images. A minimum tissue size of 40,000 μm^2^ was applied in order to eliminate artefacts from the analysis. Areas of tissue below this threshold were ignored. We also assessed breast biopsy composition in samples from the previous CER study [9] for comparison.

### Assessment of adipocyte size

Adipocyte sizing was assessed using >400 cells per sample using the Definiens Tissue Studio portal (Definiens AG, Germany), using three non-serial sections from each woman, with eight fields of view per slide. This was undertaken in breast samples in the IER study and from both breast and subcutaneous abdominal adipocytes from the previous CER study [27].

### Statistics

The required sample size was estimated using assumptions from our previous study of CER [9]. It was calculated that 20 subjects would provide 80 % power to detect a five-fold reduction in mRNA for lipogenesis enzymes between baseline (TP1) and after four to five weeks of dieting on the morning immediately after the two-day restriction (TP2). We aimed to recruit 25 participants to allow for problems with dietary compliance, insufficient tissue collected at biopsy and poor-quality RNA.

Data at the three time points are presented as mean (SD) or geometric mean (range) for log-transformed variables (insulin, insulin resistance and tryglyceride), or median (95 % CI) for non-parametric variables (oestradiol and progesterone). We assessed changes between TP1 (baseline) and TP2 (immediately after the two restricted days), TP1 and TP3 (at the end of the final week after five further unrestricted days) and between TP2 and TP3 using the the paired *t* test for parametric variables and the Wilcoxon signed rank test for non-parametric variables. Statistical significance was accepted at *p* = 0.05.

Changes in metabolic profiles were assessed by both multivariate and univariate methods. Multivariate analysis was conducted to determine the degree of separation between metabolic profiles at the three time points by integrating all four metabolomic datasets, i.e. LCMS+ LCMS–, GCMS serum and GCMS urine. This was performed using multi-block multi-level partial least squares for discriminant analysis (MB-ML-PLS-DA). The MB-ML-PLS-DA model was validated by using a bootstrapping procedure as described previously [28–30].

Univariate analysis was conducted to identify specific changes in metabolites at the three time points in two steps. First, the Friedman test [31] was applied to each metabolomics dataset to detect which metabolite(s) had significantly changed between an*y* two time points monitored. Metabolites with a *p* value <0.05 and with an FDR of *q* < 0. 1 for GCMS serum and urine were identified as statistically significant; a more conservative FDR was applied for LCMS+ *(q* < 0.01) and LCMS– (*q* < 0.05) to limit the number of significant features in these much larger datasets [32]. These significant metabolites were subsequently tested using the Tukey-Kramer test between two specific time points [33]. Metabolomics data were analysed using MATLAB 2012a (Mathworks, MA, USA), and other data using SPSS version 15 (SPSS Inc., US).

## Results

### Characteristics of the subjects

Eight hundred and thirty-three women under surveillance at the Manchester Family History Clinic received a mailed invitation to join the study. Of these, 548 did not respond and of those who did, 148 were not eligible and 113 declined. Twenty-four women agreed to take part in the study. Twenty-three completed the study and had changes in metabolomics and biochemistry assessed. The mean age of entry for completers was 40.3 (3.2) years, mean BMI was 28.1 (3.1) kg/m^2^, and mean adult weight gain since the age of 20 years was 15.0 (7.0) kg. All women had a lifetime risk of breast cancer ≥17 % [34] and were mainly Caucasian (96 %) and parous (78 %). Sufficient quality and quantity of RNA for gene expression profiling was only available for both pre- and post-intervention samples from 20 women. The 20 women in the breast gene expression analysis were not significantly different to the 23 in the whole cohort (age 40.6 (3) years, BMI 28.3 (3.1) kg/m^2^ and adult weight gain since the age of 20 years 15.2 (8.0) kg, *p* > 0.05).

### Intermittent energy restriction

Women followed the IER diet for one menstrual cycle (median 29 days) with good compliance. Twelve women had completed eight restricted days (four weeks of IER) and eleven completed ten restricted days (five weeks of IER). The degree of energy restriction during the two-day periods averaged 65 % (Fig. 1 and Additional file 1: Table S1). Subjects were asked to eat a standard Mediterranean diet at their normal energy intake during the remaining five days of the week. However, as reported in our previous studies, there was a ‘carry-over effect’ where women naturally restricted to an average of 38 % below their baseline intake on these five days. Overall energy restriction during the trial period was therefore 45 % and not the 25 % women were advised to undertake.

There were significant reductions in weight and body fat with the four to five weeks of IER when assessed at TP2 immediately after the restricted days, and five days later after the unrestricted days (TP3) (all p < 0.001) (Table 1). The apparent decrease in fat and increase in weight and FFM between TP2 and TP3 most likely reflects an increase in glycogen stores and body water which will increase the impedance-derived FFM measurement. Reductions in waist, hip and bust were comparable indicating loss from several fat depots (Table 1). There was no change in activity levels during the study period. Reported median (95 % CI) moderate activity with the IPAQ questionnaire was 140 (108–226) minutes per week at baseline TP1 and 90 (79–207) minutes at TP2 (*p* = 0.096, paired Wilcoxon test).

**Table 1:** Pairwise changes in body composition, hormones and lipids with intermittent energy restriction (IER) (n = 23 women)

	TP1^a^	TP2^a^	*P* ^1^	TP3^a^	*P* ^2^	*P* ^3^
Weight, kg	76.3 (9.1)	73.0 (9.1)	<0.001	73.1 (9.4)	<0.001	0.019
Body fat, kg	27.9 (5.5)	25.8 (5.7)	<0.001	25.4 (5.4)	<0.001	0.080
Body fat, %	36.7 (3.9)	35.5 (4.4)	<0.001	34.8 (4.4)	<0.001	0.007
Fat-free mass, kg	47.8 (4.8)	46.3 (4.7)	<0.001	47.1 (4.9)	0.002	0.001
Waist, cm	95.6 (6.5)	92.0 (7.5)	<0.001	-	-	-
Hips, cm	108.1 (5.9)	105.3 (6.1)	<0.001	-	-	-
Bust, cm	100.6 (7.5)	97.7 (7.5)	<0.001	-	-	-
Glucose, mmol/L	4.9 (0.4)	4.7 (0.3)	0.022	4.8 (0.5)	0.2	0.654
Insulin, mU/ml	8.2 (3.3–24.1)^b^	5.5 (2.2–17.1)^b^	<0.001	7.2 (2.7–16.4)^b^		0.030
HOMA, mU/mmol/l	1.8 (0.7–5.2)^b^	1.2 (0.5–3.9)^b^	<0.001	1.5 (0.6–3.4)^b^	0.04	0.036
Cholesterol, mmol/L	5.2 (0.7)	4.6 (0.7)	<0.001	4.5 (0.8)	<0.001	0.096
Triglycerides, mmol/L	1.0 (0.5–2.7)^b^	0.8 (0.5–1.8)^b^	0.007	0.9 (0.5–1.9)^b^	0.061	0.150
HDL, cholesterol mmol/L	1.4 (0.3)	1.3 (0.3)	<0.001	1.3 (0.2)	0.001	0.623
LDL, cholesterol mmol/L	3.3 (0.6)	3.0 (0.7)	0.001	2.8 (0.7)	<0.001	0.020

a Mean (+SD) or b geometric mean (range) for values. Mean (SD) *TP1* time point 1 at baseline, *TP2* time point 2 after four to five weeks of the diet immediately after the two restricted days, *TP3* time point 3 after four to five weeks of the diet after five days of ‘normal’ diet. *P* ^*1*^ paired *t* test comparing TP1 to TP2. *P* ^*2*^ paired *t* test comparing TP1 to TP3. *P* ^*3*^ paired *t* test comparing TP2 to TP3, *HOMA* homeostatic model assessment, *HDL* high-density lipoprotein, LDL low-density lipoprotein.

### Changes in indicators of systemic metabolism assessed in restricted and unrestricted phases of IER

#### Hormones and lipids

As expected, the most marked reductions in insulin, insulin resistance (HOMA) and all measured lipids occurred between baseline (TP1) and immediately after the final two day restriction (TP2) (Fig. 1). There were significant increases in levels of insulin and HOMA and reductions in LDL cholesterol between TP2 and TP3, i.e. between values immediately after the two restricted days and five days later after five unrestricted days of the diet (all p < 0.05). However, HOMA and all lipids remained significantly reduced from TP1 at TP3, i.e. after four to five weeks of the diet when measured after the unrestricted phase of IER (all *p* < 0.05) (Table 1). For women sampled in the follicular phase (n = 19), baseline (TP1) assessments were on average on day 8 of the menstrual cycle and TP2 assessments on day 11. For women sampled in the follicular phase (n = 19), baseline (TP1) assessments were on average on day 15 of the menstrual cycle and TP2 assessments on day 17. Both measurements were either consistently in the follicular phase or consistently in the luteal phase of the menstrual cycle. Differences in day of cycle at TP1 and TP2 were associated with modest increases in serum oestradiol and progesterone between the TP1 and TP2 (respective Wilcoxon signed rank test (p = 0.016 and *p* = 0.006)). The median (interquartile range) for oestradiol in women sampled in the follicular phase (n = 19) at TP1 was 482 (187–798) pMol/L and at TP2 it was 637 (474–1181) pMol/L, and for women sampled in the luteal phase (n = 4) at TP1 it was 310 (254–610) pMol/L and at TP2 it was 312 (151–491) pMol/L. The median (interquartile range) for progesterone in women sampled in the follicular phase (n = 19) at TP1 was 0.9 (0.9–1.0) nmol/L and 1.5 (1.0–3.3) nmol/L at TP 2, and for women sampled in the luteal phase (n = 4) at TP1 it was 3.6 (0.9–13.3) nmol/L and at TP2 it was 12 (1–27) nmol/L.

### Changes in the serum and urine metabolites

**Fig. 2:**
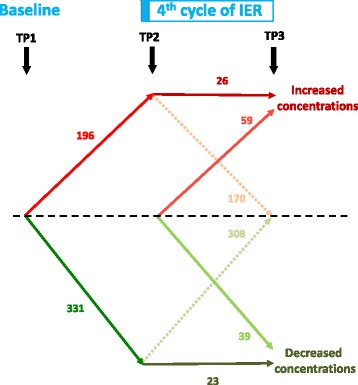
Significantly changed identified metabolites between baseline (time point 1 (*TP1*)) and during the fourth or fifth cycle of intermittent energy restriction (IER), after two days of 65 % energy restriction (*TP2*) and after 5 days of normal eating (*TP3*). Many metabolites were not significantly different from baseline at TP3 although some remained elevated. Some metabolites only increased or decreased during the five-day normal diet period between TP2 and TP3

Unique metabolic features/species were detected in the LCMS+ (n = 8100), LCMS– (n = 2208), GCMS urine (n = 190) and GCMS serum samples (n = 102). The MB-ML-PLS-DA model was able to integrate all four datasets and separate the combined metabolic profiles between TP1, TP2 and TP3. The average overall predictive accuracy was 93.0 %. The average misclassification rate between TP1 and TP2 was 0.3 %, between TP1 and TP3 it was 5.1 % and between TP2 and TP3 IT was 4.7 %. Of the total 10,600 metabolites detected by LCMS and GCMS, 1324 (12.9 %) significantly changed between one or other of the three time points (TP1 to TP2, TP1 to TP3 and TP2 to TP3) with a *p* value from the Friedman test <0.05 and below the assigned FDR for that dataset (*q* < 0.1 to *q* <0.01). Of the 1324 significant changes, 620 (53 %) had an ID. The number of identified metabolites that significantly changed between each of the time periods is shown in Fig. 2. The greatest change was between TP1 and TP2 immediately after the two days of 65 % energy restriction, where 196 metabolites significantly increased and 331 significantly decreased. The majority of these metabolites (478/527; 91 %) returned to baseline at TP3 after five days of normal eating, and thus, we assume that these are related to the acute effects of two days of 65 % energy restriction and not the cumulative effect of four to five weeks of IER. The remaining 49 metabolites (11 %) did not return to baseline between TP2 and TP3. Small numbers of metabolites increased (n = 59) or decreased (n = 39) between TP2 and TP3 when subjects were following a moderately energy-restricted Mediterranean diet (–38 % energy restriction).

Key changes in identifiable metabolites between TP1, TP2 and TP3 from univariate analysis of LCMS+ and GCMS serum and urine are summarised in Tables 2 and 3. The main acute changes between TP1 and TP2 at the end of the two-day restriction were an increase in the ketone 3-hydroxybutyric acid (GCMS urine) and acylcarnitines (LCMS+), reduced tricarboxylic acid cycle (TCA) metabolites succinic and aconitic acid (GCMS serum) reduced ubiquinol (LCMS+) and reductions in the amino acids alanine, glutamic acid, tyrosine (and associated metabolite tyramine) and increases in beta-aminoisobutyric acid (GCMS urine). This indicates increased fat oxidation, and reduced glycolysis, TCA activity, and mitochondrial electron chain flux after the two days of 65 % energy restriction. For each lipid class some metabolites increased and some decreased between TP1 and TP2. The main long-term effects of the diet between TP1 and TP3 were increased glycerolipids and unclassified lipids (LCMS+) and reductions in the amino acids glutamic acid, tyrosine (and associated metabolites tyramine and 3-p-hydroxyphenyllactic acid) (GCMS serum). Serum LCMS– data backed up the findings with LCMS+ but added little new information. The fold changes in metabolites were relatively modest. However, there was more than four-fold increase in 3-hydroxybutryic acid between TP1 and TP2, which decreased to a similar extent between TP2 and TP3. Full details of the four individual metabolomics datasets are provided in Additional file 1: Tables S2a-d.

**Table 2:** Changes in lipids and small molecule metabolites from serum liquid chromatography mass spectrometry-positive (LCMS+) mode with intermittent energy restriction (n = 23 women)

Lipid class	Biological role	Metabolites, *n*	Global change T1 ➔ T2	Global change T1 ➔ T3	Global change T2 ➔ T3
Glycerolipid (DG)	Major constituent of adipose tissue	12	4 Down	0 Down	0 Down
			1 Up	7 Up	8 Up
			7 NS	5 NS	4 NS
Fatty acid lipid (FA)	Complex lipid building block	30	23 Down	0 Down	0 Down
			2 Up	2 Up	25 Up
			5 NS	28 NS	5 NS
Cholesterol-based	Steroid precursor/cell membrane integrity	7	6 Down	0 Down	0 Down
			0 Up	0 Up	7 Up
			1 NS	7 NS	0 NS
Phosphatylcholine lipid (PC)	Major structure lipid in cell membranes	52	37 Down	1 Down	6 Down
			10 Up	2 Up	36 Up
			5 NS	49 NS	10 NS
Phosphatidylethanolamine lipid (PE)	Role in the release of lipoproteins in the liver	6	1 Down	0 Down	3 Down
			5 Up	0 Up	1 Up
			0 NS	6 NS	2 NS
Phosphatidylserine lipid (PS)	Role in cell signalling and apoptosis	1	1 Down	0 Down	0 Down
			0 Up	0 Up	0 Up
			0 NS	1 NS	1 NS
Sphingolipid (sm)	Cell surface protectant, cell signalling and recognition	15	8 Down	0 Down	0 Down
			1 Up	3 Up	11 Up
			6 NS	12 NS	4 NS
Phosphatidic lipid (PA)	Complex lipid building block and signalling	1	1 Down	0 Down	0 Down
			0 Up	0 Up	1 Up
			0 NS	1 NS	0 NS
Unclassified lipid (ul)	Various	76	31 Down	3 Down	24 Down
			39 Up	11 Up	27 Up
			6 NS	62 NS	25 NS
Small molecules	Various	57	26 Down	5 Down	17 Down
			15 Up	6 Up	31 Up
			16 NS	46 NS	9 NS

*TP1* time point 1 (baseline), *TP2* after four to five weeks of the diet immediately after the two restricted days, *TP3* after four to five weeks of the diet after five days of normal eating, *NS* no significant change

**Table 3:** Main metabolite changes with intermittent energy restriction (IER), on gas chromatography mass spectrometry (GCMS) in serum and GCMS in urine with IER (n = 23)

Metabolite	Global change TP1 ➔ TP2	Global change TP1 ➔ TP3	Global change TP2 ➔ TP3
GCMS serum			
Fat oxidation and ketogenesis			
Butanoic acid/butyric acid	Up	NS	Down
3-Hydroxybutyric acid	Up	NS	Down
Glycerol	Up	NS	Down
Hexadecanoic acid	Up	NS	Down
Linoleic acid	Up		Down
Amino acids			
Tyramine (tyrosine metabolite)	Down	Down	NS
Glutamic acid	Down	Down	NS
p-Hydroxyphenyllactic acid	NS	Down	NS
GCMS urine			
Amino acids			
Tyrosine	Down	Down	NS
Alanine	Down	NS	NS
TCA metabolites			
Succinic acid	Up	NS	NS
Aconitic acid	Up	NS	NS
Myokine or muscle breakdown product			
Beta-aminoisobutyric acid	Up	NS	NS

*TP1* time point 1 (baseline), *TP2* after four to five weeks of the diet immediately after the two restricted days, *TP3* after four to five weeks of the diet after five days of normal eating, *NS* no significant change

### Effect of IER on breast and lymphocyte gene expression

**Fig. 3:**
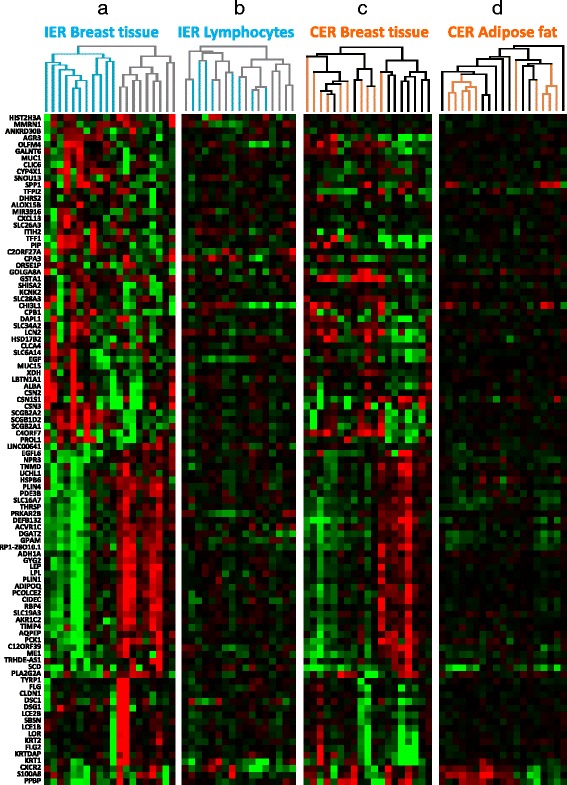
Unsupervised analysis of the most changed genes in the breast in participants following intermittent energy restriction (*IER*) and continuous energy restriction (*CER*) compared with changes in peripheral blood lymphocyctes and abdominal fat. The 100 genes with the highest variance in changed gene expression in breast tissue following IER are shown in **a**. The same genes in the lymphocytes from IER participants (**b**), the breast in women undertaking CER (**c**) and subcutaneous abdominal fat (**d**) in women undertaking CER are shown for comparison. Subjects on IER (*blue*) represent responders, *grey* represents non-responders. Participants on CER are shown in orange and non-dieting controls in *black*. The heatmap shows relative paired log2 changes in gene expression (after compared to before), *green* = downregulation, *red* = upregulation, *black* = no change. In the IER responders there was downregulation of many metabolic genes similar to women undertaking CER. In the IER responders some genes are also upregulated. The IER non-responders had expression profiles similar to women who were non-diet controls (*black* in **c**). The 100 genes with the highest variance were only minimally changed in peripheral blood lymphocytes (**b**) or subcutaneous abdominal fat (**d**)

The area of fat, stroma and epithelium in biopsy pairs was not significantly different between TP1 (35 % fat, 55 % stroma and 10 % epithelium) and TP2 (40 % fat, 52 % stroma and 8 % epithelium) samples (*p* = 0.30, Mann-Whitney test). It was not possible to identify any significantly differentially expressed genes between the breast biopsies taken before and after IER in a pairwise manner using pairwise SAM as found in our previous study [9]. A possible explanation for the lack of consistency between participants was that changes in expression were highly variable between participants. To examine this further, we performed unsupervised hierarchical cluster analysis of the 100 genes with the highest variance of change in expression across the 20 participants (Fig. 2) and identified two groups of participants: a group with breast tissue which appears to respond to IER (green) (n = 11) with expected downregulation of metabolic genes associated with energy restriction, i.e. reduced lipid biosynthesis, gluconeogenesis and glycogen synthesis, and a group with breast tissue that does not appear to respond (grey) (n = 9). Some of the responders also had increased expression of genes associated with breast epithelial cell differentiation (milk proteins, secretoglobulins and mucins). Other transcripts with highly variable changes in expression in the breast tissue after IER included leptin, adiponectin, chemokines, epidermal growth factor (EGF) and ACVR1C receptors, heat shock protein and reduced collagen synthesis and breakdown (Fig. 3a). These changes appeared to be breast-specific and were not seen in the gene expression profiles of peripheral blood lymphocytes taken at the same intervals (Fig. 3b).

If lymphocytes are to be used as a reliable surrogate for breast tissue gene expression, there should be much stronger correlation between the samples from the same individual than between non-paired samples. However, changes between matched breast and lymphocyte samples from the same participants were not more strongly significantly correlated more strongly compared to un-matched samples. Pairwise analysis of changes in expression of the lymphocytes did not identify any genes that were consistently significant using the siggenes package with a 5 % FDR.

### Comparisons between responders and non-responders

We undertook diagnostic analyses to assess whether the apparent differences in breast gene expression in response to IER could be an artefact linked to different composition of the breast biopsies, i.e. the proportion of fat, stroma and epithelium or differences in the day of cycle or serum oestradiol and progesterone levels between the pre and post intervention biopsies. We also assessed whether differences were linked to baseline characteristics, breast density (measured visually and volumetrically) and change in weight, insulin, lipids, breast adipocyte size, or the metabolome in serum or urine in the different groups. There were no significant differences in any of these parameters (Additional file 1: Table S3). There were no differences in the metabolome between responders and non-responders on MB-ML-PLS-DA multivariate analyses. Univariate analyses were inconclusive and only identified differences in 20 serum (LCMS+) and three urine (GCMS) metabolites, which respectively represent 0.3 % and 1.6 % of the unique serum and urine metabolites (data not shown).

These data suggest that the difference in responsiveness of breast tissue is not explained by methodological issues. Differences between women appear to be breast-specific and not obviously related to the phenotype of the women or systemic changes in weight or hormones (e.g. insulin) or other potential metabolic mediators found in the serum or urine metabolome.

### Comparison of the effects of IER (two days 65 % energy restriction per week) to the effects of 60 % CER for one menstrual cycle on breast gene expression, adipocyte size and metabolomic changes

Reductions in total body fat with IER were less than those previously seen with CER: –7.96 (SD 5.04) vs. –11.23 (SD 3.78) %, which is consistent with the lesser overall energy restriction of 45 % with IER vs. 60 % with CER (Additional file 1: Table S4). Reductions in insulin, lipids and metabolite levels detected during the IER, i.e. reduced amino acids and increases in tricarboxylic acid metabolites and ketone bodies, were consistent with those previously seen with CER (3614 kJ, 58 g protein and 97 g carbohydrate per day) [9].

**Fig. 4:**
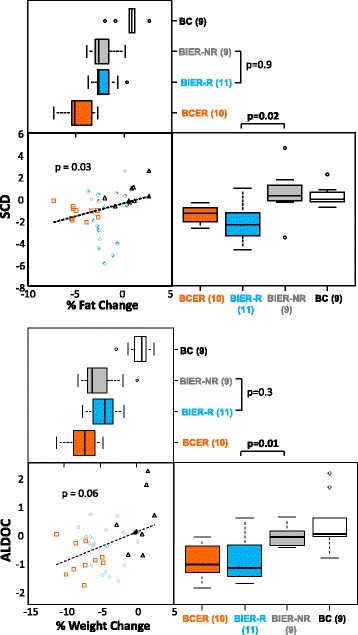
Changes in weight and body fat are correlated with transcriptional markers of energy restriction in breast tissue, but only half of the participants on intermittent energy restriction (*IER*) have expected molecular changes. Examples demonstrating that IER induces more subtle and variable changes than continuous energy restriction (*CER*) in breast tissue, both in terms of fat and weight percentage changes and changes in transcription of stearoyl-CoA desaturase (*SCD*) (fat synthesis) and aldolase C, fructose-bisphosphate (*ALDOC*) (glycolysis). Transcriptional changes similar to those seen with CER are limited to the IER responders (*R*, *blue*, diamonds), rather than non-responders (*NR*, *grey*, *diamonds*) identified in Fig. 2. *BIER* breast intermittent energy restriction, *BCER* breast continuous energy restriction (*orange*, *squares*), *BC* breast non-dieting controls (*white*, *black triangles*). For correlation the *p* values are for Spearman’s correlation analysis and group-wise *p* values are for the Wilcoxon test

Changes in energy-restriction-associated gene expression in the 11 responding IER subjects were comparable to those observed across all the participants in the previous 60 % CER study with 9/10 of the CER participants clustering together. The expression profiles of non-responders in the IER study appeared similar to the non-dieting controls in the earlier CER study (Fig. 3a, c) and was confirmed by gene set enrichment analysis (adjusted *p* = 0.027). None of the changes in breast gene expression with CER were seen in lymphocytes in the IER study (Additional file 2: Figure S1). In the previous CER study, 71 probe sets (representing 65 genes) were significantly changed in the breast tissue of 10 participants after four to five weeks of 60 % CER. Using this gene set as a molecular classifier and applying it to predict the status of the IER cases using the PAM method [25] resulted in a similar dichotomy to the unsupervised analysis shown in Fig. 3, with 8 out of 20 participants predicted to have undergone energy restriction (Additional file 2: Figure S1). Overall we found that IER induces more subtle and variable changes on breast gene expression than CER. The response of changes in stearoyl-CoA desaturase (SCD) and aldolase C, fructose-bisphosphate (ALDOC) in breast tissue across the two studies are reported in Fig. 4. Notably the variable response of these metabolic genes with IER is not related to changes in body fat or weight; we could speculate that this may be due to genetic differences between participants, but do not have any direct evidence.

The previous CER study included abdominal subcutaneous fat and breast biopsies. Very few of the 100 most variable gene changes in the breast with CER were also highly variable across abdominal fat, suggesting breast specificity (Fig. 3d). In the CER study, baseline subcutaneous abdominal fat adipocytes were significantly larger than breast adipocytes (4066 μm^2^ vs. 3194 μm^2^*p* < 0.0001). There was a highly significant decrease in subcutaneous abdominal adipocyte size with four to five weeks of 60 % CER of 4066 μm^2^ to 3593 μm^2^ (–11 %; *p* < 0.0001), which occurred in association with an 11 % reduction in total body fat. In contrast there was a small numerical reduction in mean breast adipocyte size in response to CER, which was not statistically significant (3194 μm^2^ to 2989 μm^2^ –6.4 %, p > 0.121). There was a small, statistically significant reduction in mean breast adipocyte size from 2221 μm^2^ to 2087 μm^2^ (–6.0 %, *p* < 0.01) post IER alongside a 7 % reduction in body fat (*p* < 0.01).

The baseline breast adipocyte size was smaller in the IER compared to the CER samples (2221 μm^2^ vs. 3194 μm^2^, *p* < 0.0001), which may be linked to the lower body fat levels in the IER group (36.7 ± 3.9 % vs. 44.0 ± 2.3 % body fat (Additional file 1: Table S4)) or the larger amount of stroma in the IER samples [35]. A linear regression of the combined IER and CER datasets including both these factors explained 38 % of the variation in fat cell size. The trial in which the subject was participating was a significant predictor in this model (*p* = 0.035) whereas percentage body fat was not (*p* = 0.455). There were no significant differences in changes in fat cell size between responders and non-responders to IER (Additional file 1: Table S3).

## Discussion

This is the first study to examine the effects of IER on the breast and systematic metabolism. All women had marked changes in weight, adiposity, systemic hormones, lipids and metabolites. However, only half of the women had breast gene expression changes associated with decreased anabolism, i.e. decreased fatty acid biosynthesis, glycolysis, gluconeogenesis and glycogen synthesis. The inconsistent breast tissue gene expression changes with IER did not correlate with compliance, or the changes in weight or systemic metabolism, thus appear to be participant-specific. This requires further study to determine the underlying mechanism. The changes in metabolic gene expression profiles in the 11 responders to IER resembled changes in women undertaking a 60 % CER for one menstrual cycle from our previous study whilst the 9 non-responders resembled the non-dieting controls in this study [9]. However, we acknowledge that differences in entry criteria and sample processing between the studies may have affected these results. Some of the women following IER also appeared to have had increases in epithelial cell differentiation (i.e. increased lactalbumin caseins, mammoglobins and mucins) and decreases in collagen synthesis (TNMD, PCOLCE2, TIMP4), which was less evident in women on CER and may be specific to IER.

We studied IER because previous short-term randomised trials in women have reported greater reductions in weight and adiposity [10, 11] and insulin [11] compared with CER. Part of the reported superiority of IER may be because of greater energy restriction than CER [10, 11]. In the study reported here the two-day IER was designed to produce an overall 25 % ER, but a carry-over effect after the two-day restriction (average 38 % reduction) meant that intake was also reduced during the next five days of the standard Mediterranean diet. Thus, the participants undertook an overall 45 % energy restriction rather than the projected 25 %. Our findings with IER do not necessarily inform what would happen with just two days of restriction and five days of completely normal intake on the other days of the week. This pragmatic study is instead reporting the breast and systemic effects of a two-day IER regimen, with the magnitude of restriction that we have found occurs with this pattern of eating [10, 11].

IER induced large fluctuations in insulin, with the greatest reductions seen after the two 65 % energy restriction days (–30 % from baseline) and lesser reductions on unrestricted days (–10 % from baseline) as shown previously [10, 11]. Likewise, IER induced large changes in metabolites after the two restricted days, which reflects a switch to fat oxidation and reduced glycolysis and TCA activity. The two-day restriction was also associated with increased urine levels of beta-aminoisobutyric acid and increased TCA metabolites, which have both been linked to improved mitochondrial function and insulin sensitivity and reduced risk of diabetes, obesity and cardiovascular disease [36–38].

Interestingly the potentially beneficial changes in metabolism reported in the restricted days of IER were not seen after the five days of the moderately energy-restricted (38 %) Mediterranean diet with IER. A 38 % energy restriction is at the limits of what can be achieved with standard continuous energy-restricted diets (typically 25–40 %). Thus, this raises the possibility that standard daily dieting may not elicit shifts in metabolism and increased fat oxidation, which can only be achieved during spells of a more severe restriction (≥65 %) with IER.

Whether such profound cyclical changes in the serum metabolites between restricted and non-restricted days are beneficial or harmful to health is not known, The ability to switch from fat oxidation in an energy-deficient state and carbohydrate metabolism in a post-prandial state is considered important for metabolic health and to reduce the risk of obesity-related diseases such as cancer and cardiovascular disease [39]. IER also led to sustained reductions in the amino acids glutamic acid and tyrosine during the restricted and the normal healthy eating days. These reductions may indicate improved insulin sensitivity and reduced muscle proteolysis or increased oxidative metabolism of amino acids [40].

IER was originally introduced in rodent experiments to make overall energy restriction easier to administer [41]. Subsequent rodent studies indicate IER is equivalent or superior to CER in terms of weight loss, improving insulin sensitivity, preventing tumours, increasing resistance to neuronal damage, reducing cognitive impairment, protecting the heart and increasing the lifespan of rodents [5]. However, the safety of IER in the long term is not established, even in rodents. The metabolomics data link IER to marked acute increases in ketones and some fatty acids. There are potential concerns that a rise in circulating fatty acids could promote tumour growth [42]. Ketones have been related to both reduction and increases in tumour cell proliferation and growth [43, 44]. The ketone sodium butyrate has been linked to increased breast cell differentiation as assessed morphologically and by lactalbumin production [45, 46], which may have a role in the increased expression of differentiation genes we reported in some of the patients on IER.

Gene expression changes in lymphocytes were not consistent with changes in the breast, and so are not likely to be a useful biomarker of changes in the breast with energy restriction. In contrast, previous dietary intervention studies have found that lymphocytes can reflect liver and adipose transcriptomics and are useful for studying genes related to fatty acid and cholesterol metabolism and inflammation [47].

This is the first study to report changes in breast adipocyte cell size with energy restriction and weight loss in humans. Breast adipocytes were smaller than abdominal adipocytes amongst the obese subjects studied in our previous CER trial. This contrasts to previous reports of comparable fat cell size and lipolytic responses between breast and subcutaneous abdominal fat cells amongst normal and overweight subjects [48]. The CER group experienced significant reductions in abdominal fat size (–11 %) with weight loss as previously documented [49, 50]. Reduced fat cell size is believed to mediate improved insulin sensitivity rather than reductions in fat cell numbers [51], and is likely to account for the reduced abdominal fat mass. Reductions in breast fat indicated by reduced bust measurements with CER did not occur alongside significant reductions in breast adipocyte size suggesting that reduced fat in the breast may result from reduced fat cell numbers, rather than reduced fat cell size. The modest reduction in breast adipocyte size with IER (6 %) alongside a 7 % reduction in total body fat is likely to reflect acute lipolysis on the preceding two energy-restricted days. The failure of CER and IER to significantly impact on breast adipocyte size in the premenopausal at-risk breast is intriguing. Large breast adipocytes have been found to be associated with increased inflammation and aromatase expression, mainly amongst obese postmenopausal women [52]. Further data on weight loss on breast adipocyte size and number in premenopausal and postmenopausal women are required.

Changes in breast and systemic metabolism seen after four to five weeks of IER were brought about by effects of diet-induced weight loss, reduced energy intake or modifications of diet composition, i.e. reductions in energy, carbohydrate, fat and protein with IER. There were no reported increases in physical activity. Pre and post study IPAQ physical activity scores both reflect relatively high levels of activity but are assumed to be overestimates, which is well-known with physical activity questionnaires [53]. Future trials should assess physical activity using direct assessment methods such as accelerometry.

The strength of this study is the good compliance with the IER with completion of restricted days immediately prior to post-diet assessments in all subjects and near complete assessment of study endpoints amongst our cohort. Weaknesses include the relatively small numbers of subjects and lack of a direct randomised comparison to a non-diet or CER group. Some of our conclusions depend on case study comparisons between the current IER and a previous CER study. Women in both the IER and CER cohorts had regular cycles, were premenopausal, and at increased risk of breast cancer, but there were key differences between the groups. Women in the CER study had a greater starting weight than the IER cohort (mean (SD) BMI 33.9 (3) kg/m^2^ vs. 28.1 (3.1) kg/m^2^, *p* < 0.001 independent samples *t* test). In addition, CER breast biopsies were not targeted to stroma and thus, had a higher fat content compared with the IER biopsies; CER (87 % fat, 10 % stroma and 3 % epithelium) vs. IER (35 % fat, 55 % stroma and 10 % epithelium) (*p* < 0.001 Mann-Whitney test).

We aimed to standardise biopsy and metabolic measurements in the first week of the menstrual cycle to avoid confounding hormonal effects. This proved difficult within our cohort due to clinical logistics. On average women were reassessed three to four days later in their cycle than baseline assessments, but remained in either the follicular or luteal phase and this did not explain the gene expression responder and non-responder groups identified.

## Conclusions

In conclusion we have demonstrated that IER is associated with marked weight loss and reductions of insulin and lipids. A large number of serum and urine metabolites fluctuate in the restricted and unrestricted phases of the diet. IER by a 65 % energy restriction on days per week (overall 45 % energy restriction) had a variable effect on breast gene expression. Only half of the participants had gene expression evidence characteristic of anabolism (downregulation of lipogenesis and glycolysis), whereas a more consistent response was observed with a continuous 60 % energy restriction. Further investigations of the effects of an intermittent energy restriction (two days/week of 65 % energy restriction) and identifying women who may respond to this pattern and level of energy restriction are warranted, as this represents an effective and potentially feasible method of energy restriction.

## Abbreviations

ALDOC aldolase C, fructose-bisphosphate; BMI, body mass index; CER, continuous energy restriction; DAVID, Database for Annotation, Visualization and Integrated Discovery; FDR, false discovery rate; GCMS, gas chromatography mass spectrometry; HDL, high-density lipoprotein; HOMA, homeostatic model assessment; IER, intermittent energy restriction; LCMS, liquid chromatography mass spectrometry; LDL, low-density lipoprotein; MB-ML-PLS-DA, multi-block multi-level partial least squares for discriminant analysis; PAM, prediction analysis of microarrays; SAM, significance analysis of microarrays; SCD, stearoyl-CoA desaturase; TCA, tricarboxylic acid cycle; TP, time point

## Additional files

Additional file 1: Table S1. Dietary intake at baseline and after four to five weeks of IER, during restricted and unrestricted days of IER (n = 23). Table S2. **a** LCMS+ changes in identified serum metabolites after four to five weeks of IER, on restricted and unrestricted days of IER. **b** LCMS– changes in identified serum metabolites after four to five weeks of IER on restricted and unrestricted days of IER. **c** GCMS changes in identified serum metabolites after four to five weeks of IER on restricted and unrestricted days of IER. **d** GC-MS changes in identified urine metabolites after four to five weeks of IER on restricted and unrestricted days of IER. Table S3 Comparison of baseline characteristics, physical and metabolic changes between the molecular responders and non-responders identified by breast tissue gene expression following IER. Table S4 Percentage change in body weight, BMI, adiposity, lipid and hormone levels with four to five weeks of IER in comparison with four to five weeks of 60 % continuous energy restriction. (PPTX 233 kb) Additional file 2: Figure S1. Changes in gene expression due to CER are also changed in approximately half of the IER participants, but are not clearly correlated with lymphocytes. The heatmap colours show log2 fold change in gene expression of the baseline samples relative to the post-diet samples (*green* = downregulated, *red* = upregulated, *black* = no change). The *arrows* show subjects predicted to have an energy restriction (*orange* = CER, *blue* = IER) or unchanged profiles (*grey*). Similar results were obtained when considering all probe sets on the array. (XLSX 268 kb)

## References

[1] Harvie M, Howell A, Vierkant RA, Kumar N, Cerhan JR, Kelemen LE (2005). Association of gain and loss of weight before and after menopause with risk of postmenopausal breast cancer in the Iowa women’s health study. Cancer Epidemiol Biomarkers Prev.

[2] Eliassen AH, Colditz GA, Rosner B, Willett WC, Hankinson SE (2006). Adult weight change and risk of postmenopausal breast cancer. JAMA.

[3] Teras LR, Goodman M, Patel AV, Diver WR, Flanders WD, Feigelson HS (2011). Weight loss and postmenopausal breast cancer in a prospective cohort of overweight and obese US women. Cancer Causes Control.

[4] Huang Z, Hankinson SE, Colditz GA, Stampfer MJ, Hunter DJ, Manson JE (1997). Dual effects of weight and weight gain on breast cancer risk. JAMA.

[5] Harvie M, Howell A (2012). Energy restriction and the prevention of breast cancer. Proc Nutr Soc.

[6] Meynet O, Ricci JE (2014). Caloric restriction and cancer: molecular mechanisms and clinical implications. Trends Mol Med.

[7] Fabian CJ, Kimler BF, Donnelly JE, Sullivan DK, Klemp JR, Petroff BK (2013). Favorable modulation of benign breast tissue and serum risk biomarkers is associated with > 10 % weight loss in postmenopausal women. Breast Cancer Res Treat.

[8] Hursting SD (2014). Obesity, energy balance, and cancer: a mechanistic perspective. Cancer Treat Res..

[9] Ong KR, Sims AH, Harvie M, Chapman M, Dunn WB, Broadhurst D (2009). Biomarkers of dietary energy restriction in women at increased risk of breast cancer. Cancer Prev Res (Phila Pa).

[10] Harvie M, Wright C, Pegington M, McMullan D, Mitchell E, Martin B (2013). The effect of intermittent energy and carbohydrate restriction v. daily energy restriction on weight loss and metabolic disease risk markers in overweight women. Br J Nutr.

[11] Harvie MN, Pegington M, Mattson MP, Frystyk J, Dillon B, Evans G (2011). The effects of intermittent or continuous energy restriction on weight loss and metabolic disease risk markers: a randomized trial in young overweight women. Int J Obes (Lond).

[12] Johnstone A. Fasting for weight loss-an effective strategy or latest dieting trend? Int J Obes (Lond). 2015;39(5):727-33. 10.1038/ijo.2014.214 25540982

[13] Schofield WN (1985). Predicting basal metabolic rate, new standards and review of previous work. Hum Nutr Clin Nutr..

[14] Ainsworth BE, Haskell WL, Herrmann SD, Meckes N, Bassett DR, Tudor-Locke C (2011). 2011 Compendium of Physical Activities: a second update of codes and MET values. Med Sci Sports Exerc.

[15] Karolinska Institutet. International physical activity questionnaire. Karolinska Institutet 2013 November 4. Available from www.ipaq.ki.se. Accessed 25 May 2016.

[16] Matthews DR, Hosker JP, Rudenski AS, Naylor BA, Treacher DF, Turner RC (1985). Homeostasis model assessment: insulin resistance and beta-cell function from fasting plasma glucose and insulin concentrations in man. Diabetologia.

[17] Dunn WB, Broadhurst D, Begley P, Zelena E, Francis-McIntyre S, Anderson N (2011). Procedures for large-scale metabolic profiling of serum and plasma using gas chromatography and liquid chromatography coupled to mass spectrometry. Nat Protoc.

[18] Begley P, Francis-McIntyre S, Dunn WB, Broadhurst DI, Halsall A, Tseng A, Knowles J, Goodacre R, Kell DB. Development and performance of a gas chromatography-time-of-flight mass spectrometry analysis for large-scale nontargeted metabolomic studies of human serum. Anal Chem. 2009;81(16):7038-46. 10.1021/ac9011599 19606840

[19] Boyum A (1968). Separation of leukocytes from blood and bone marrow. Introduction. Scand J Clin Lab Invest Suppl..

[20] Gentleman RC, Carey VJ, Bates DM, Bolstad B, Dettling M, Dudoit S (2004). Bioconductor: open software development for computational biology and bioinformatics. Genome Biol.

[21] Dai M, Wang P, Boyd AD, Kostov G, Athey B, Jones EG (2005). Evolving gene/transcript definitions significantly alter the interpretation of GeneChip data. Nucleic Acids Res.

[22] Wu Z, Irizarry RA (2004). Preprocessing of oligonucleotide array data. Nat Biotechnol.

[23] Breitling R, Armengaud P, Amtmann A, Herzyk P (2004). Rank products: a simple, yet powerful, new method to detect differentially regulated genes in replicated microarray experiments. FEBS Lett.

[24] Tusher VG, Tibshirani R, Chu G (2001). Significance analysis of microarrays applied to the ionizing radiation response. Proc Natl Acad Sci USA.

[25] Tibshirani R, Hastie T, Narasimhan B, Chu G (2002). Diagnosis of multiple cancer types by shrunken centroids of gene expression. Proc Natl Acad Sci USA.

[26] Huang DW, Sherman BT, Lempicki RA (2009). Systematic and integrative analysis of large gene lists using DAVID bioinformatics resources. Nat Protoc.

[27] Mitchell A, Clarke RB, Spence K, Bradley H, Harvie M, Howell T. A novel approach to adipocyte size quantitation. Annual Microscience Microscopy Congress of the Royal Microscopical Society; 2014 Jul 1-3; Manchester, UK. 2014

[28] Xu Y, Fowler SJ, Bayat A, Goodacre R (2014). Chemometrics models for overcoming high between subject variability: applications in clinical metabolic profiling studies. Metabolomics..

[29] Xu Y, Correa E, Goodacre R (2013). Integrating multiple analytical platforms and chemometrics for comprehensive metabolic profiling: application to meat spoilage detection. Anal Bioanal Chem.

[30] Xu Y, Goodacre R (2012). Multiblock principal component analysis: an efficient tool for analyzing metabolomics data which contain multiple influential factors. Metabolomics..

[31] Hollander M, Wolfe D A. Non parametric statistical methods. University of California USA: John Wiley and Sons; 1999.

[32] Storey JD. A direct approach to false discovery rates. J Royal Statistical Soc, Series B (Statistical Methodology). 2002;64(3):479–98.

[33] Hochberg Y, Tamhane AC. Multiple Comparison Procedures. John Wiley & Sons; 1987.

[34] Tyrer J, Duffy SW, Cuzick J (2004). A breast cancer prediction model incorporating familial and personal risk factors. Stat Med.

[35] Brown JQ, Bydlon TM, Kennedy SA, Caldwell ML, Gallagher JE, Junker M (2013). Optical spectral surveillance of breast tissue landscapes for detection of residual disease in breast tumor margins. PLoS One.

[36] Roberts LD, Bostrom P, O’Sullivan JF, Schinzel RT, Lewis GD, Dejam A (2014). beta-Aminoisobutyric acid induces browning of white fat and hepatic beta-oxidation and is inversely correlated with cardiometabolic risk factors. Cell Metab.

[37] Abu Bakar MH, Sarmidi MR, Cheng KK, Ali KA, Suan CL, Zaman HH, Yaakob H. Metabolomics - the complementary field in systems biology: a review on obesity and type 2 diabetes. Mol Biosyst. 2015;11(7):1742-74 10.1039/c5mb00158g 25919044

[38] Calderon-Santiago M, Priego-Capote F, Galache-Osuna JG, de Castro MD L (2013). Method based on GC-MS to study the influence of tricarboxylic acid cycle metabolites on cardiovascular risk factors. J Pharm Biomed Anal..

[39] Muoio DM (2014). Metabolic inflexibility: when mitochondrial indecision leads to metabolic gridlock. Cell.

[40] Seibert R, Abbasi F, Hantash FM, Caulfield MP, Reaven G, Kim SH. Relationship between insulin resistance and amino acids in women and men. Physiol Rep. 2015;3(5). 10.14814/phy2.12392 PMC4463823 25952934

[41] Carlson AJ, Hoezel F (1946). Apparent prolongation of the life span of rats by intermittent fasting. J Nutr..

[42] Nieman KM, Kenny HA, Penicka CV, Ladanyi A, Buell-Gutbrod R, Zillhardt MR (2011). Adipocytes promote ovarian cancer metastasis and provide energy for rapid tumor growth. Nat Med.

[43] Seyfried TN, Marsh J, Shelton LM, Huysentruyt LC, Mukherjee P. Is the restricted ketogenic diet a viable alternative to the standard of care for managing malignant brain cancer? Epilepsy Res. 2011;100(3):310-26. 10.1016/j.eplepsyres.2011.06.017 21885251

[44] Martinez-Outschoorn UE, Lin Z, Whitaker-Menezes D, Howell A, Sotgia F, Lisanti MP (2012). Ketone body utilization drives tumor growth and metastasis. Cell Cycle.

[45] Grieve RJ, Woods KL, Mann PR, Smith SC, Wilson GD, Howell A (1980). Effect of sodium butyrate on synthesis of specific proteins by human breast-carcinoma cells. Br J Cancer.

[46] Takahara J, Yunoki S, Yakushiji W, Yamauchi J, Yamane Y (1977). Stimulatory effects of gamma-hydroxybutyric acid on growth hormone and prolactin release in humans. J Clin Endocrinol Metab.

[47] de Mello VD, Kolehmanien M, Schwab U, Pulkkinen L, Uusitupa M (2012). Gene expression of peripheral blood mononuclear cells as a tool in dietary intervention studies: What do we know so far?. Mol Nutr Food Res.

[48] Rebuffe-Scrive M, Eldh J, Hafstrom LO, Bjorntorp P (1986). Metabolism of mammary, abdominal, and femoral adipocytes in women before and after menopause. Metabolism.

[49] Mauriege P, Imbeault P, Langin D, Lacaille M, Almeras N, Tremblay A (1999). Regional and gender variations in adipose tissue lipolysis in response to weight loss. J Lipid Res.

[50] Larson-Meyer DE, Heilbronn LK, Redman LM, Newcomer BR, Frisard MI, Anton S (2006). Effect of calorie restriction with or without exercise on insulin sensitivity, beta-cell function, fat cell size, and ectopic lipid in overweight subjects. Diabetes Care.

[51] Andersson DP, Eriksson HD, Thorell A, Toft E, Qvisth V, Naslund E (2014). Changes in subcutaneous fat cell volume and insulin sensitivity after weight loss. Diabetes Care.

[52] Iyengar NM, Morris PG, Zhou XK, Gucalp A, Giri D, Harbus MD (2015). Menopause is a determinant of breast adipose inflammation. Cancer Prev Res (Phila).

[53] Lee PH, Macfarlane DJ, Lam TH, Stewart SM (2011). Validity of the International Physical Activity Questionnaire Short Form (IPAQ-SF): a systematic review. Int J Behav Nutr Phys Act.

