# Supplementary material for: Intermittent energy restriction induces changes in breast gene expression and systemic metabolism
Source: Breast Cancer Res. 2016 May 28;18:57. doi: 10.1186/s13058-016-0714-4 (PMC4884347; doi:10.1186/s13058-016-0714-4)
Supplement: Additional file 1: Table S1. — Dietary intake at baseline and after four to five weeks of IER, during restricted and unrestricted days of IER (n = 23). Table S2. a LCMS+ changes in identified serum metabolites after four to five weeks of IER, on restricted and unrestricted days of IER. b LCMS– changes in identified serum metabolites after four to five weeks of IER on restricted and unrestricted days of IER. c GCMS changes in identified serum metabolites after four to five weeks of IER on restricted and unrestricted days of IER. d GC-MS changes in identified urine metabolites after four to five weeks of IER on restricted and unrestricted days of IER. Table S3 Comparison of baseline characteristics, physical and metabolic changes between the molecular responders and non-responders identified by breast tissue gene expression following IER. Table S4 Percentage change in body weight, BMI, adiposity, lipid and hormone levels with four to five weeks of IER in comparison with four to five weeks of 60 % continuous energy restriction. (PPTX 233 kb) [file 13058_2016_714_MOESM1_ESM.pptx]

## Slide 1
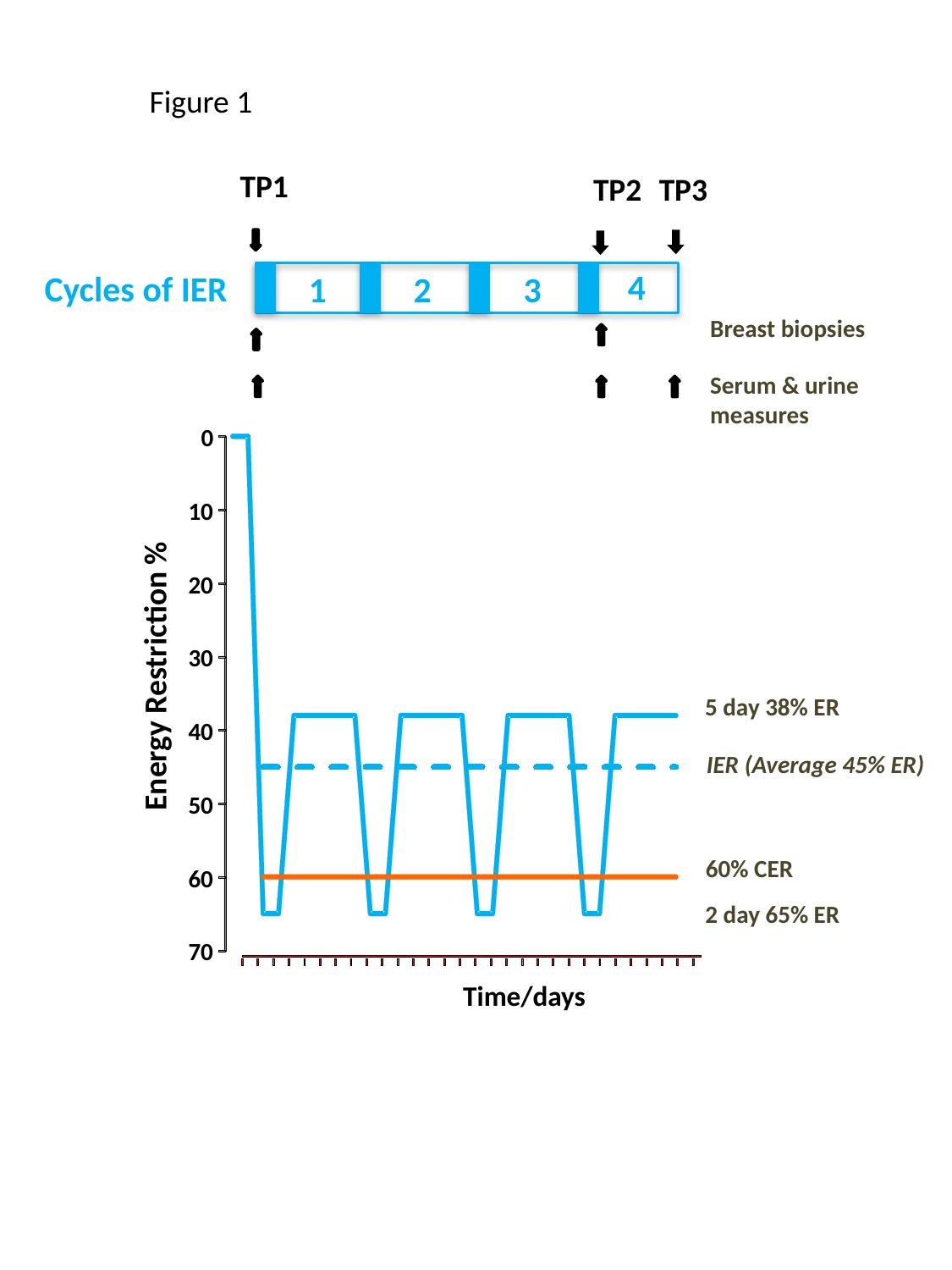

Figure 1
TP1
TP2
TP3
4
1
2
3
Cycles of IER
Breast biopsies
Serum & urine
measures
0
10
Energy Restriction %
20
30
5 day 38% ER
40
IER (Average 45% ER)
50
60% CER
60
2 day 65% ER
70
Time/days

## Slide 2
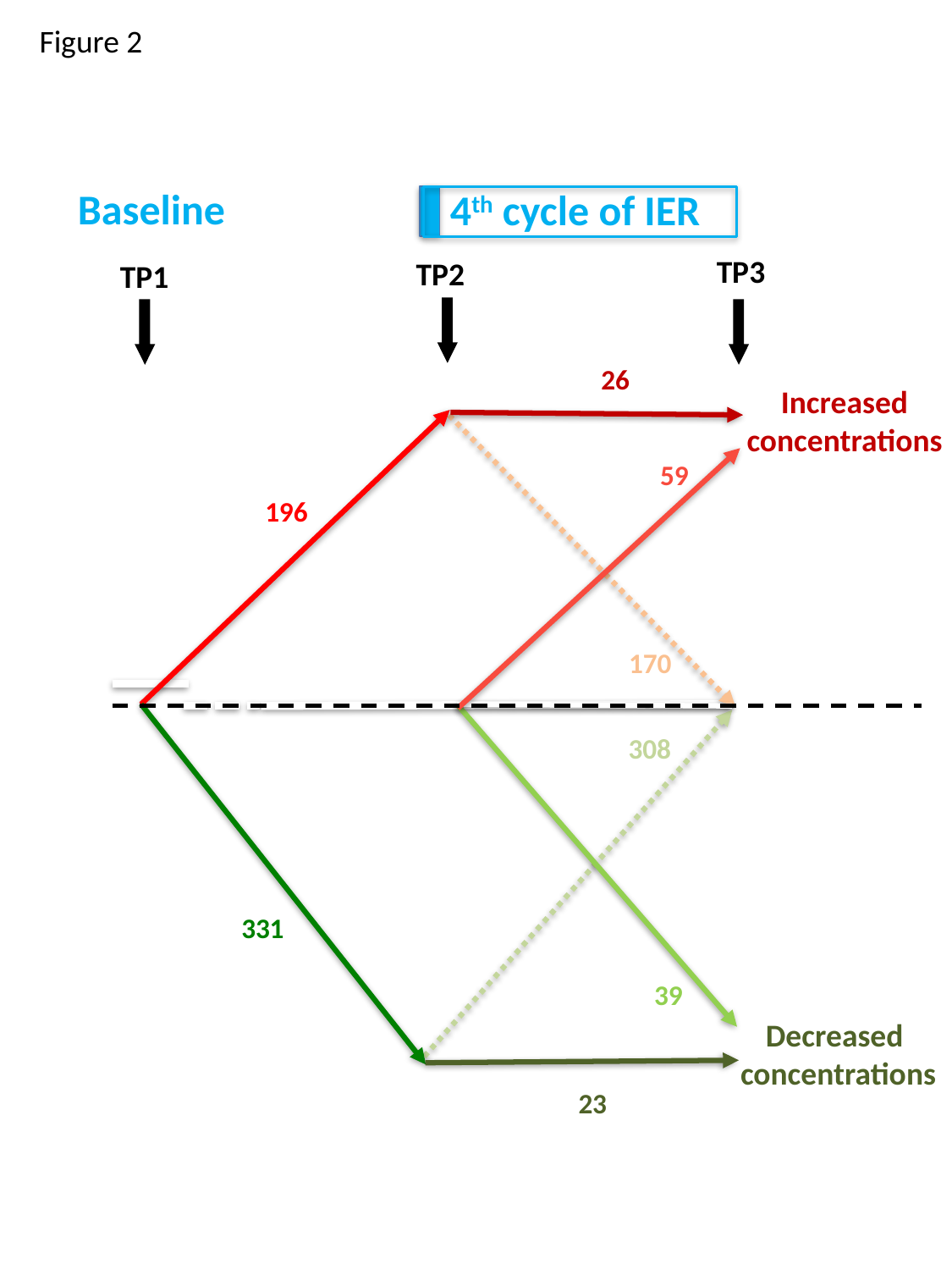

Figure 2
Baseline
4th cycle of IER
TP3
TP2
TP1
26
Increased
concentrations
59
196
170
308
331
39
Decreased
concentrations
23

## Slide 3
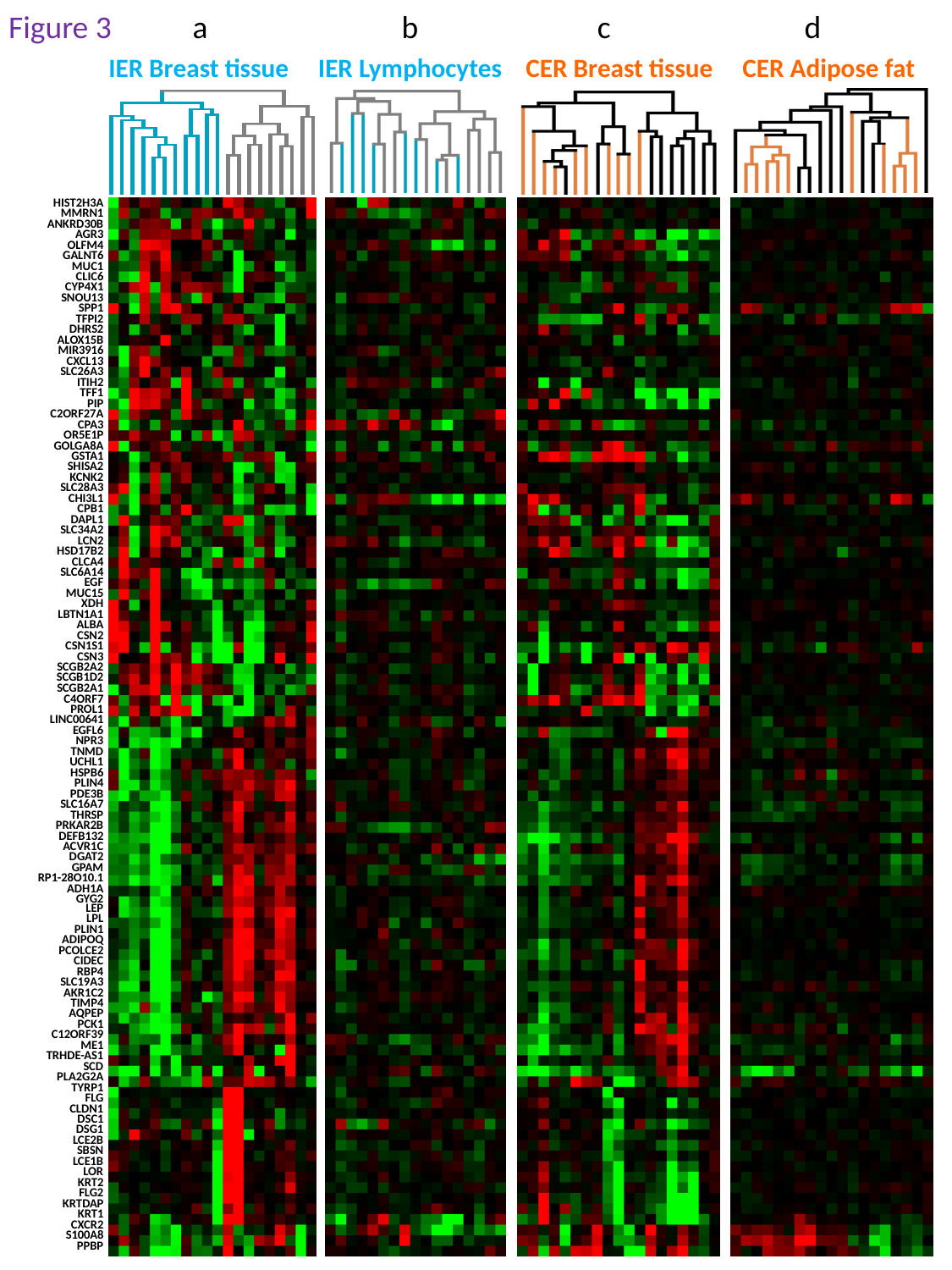

Figure 3
a b c d
IER Breast tissue IER Lymphocytes CER Breast tissue CER Adipose fat
HIST2H3A
MMRN1
ANKRD30B
AGR3
OLFM4
GALNT6
MUC1
CLIC6
CYP4X1
SNOU13
SPP1
TFPI2
DHRS2
ALOX15B
MIR3916
CXCL13
SLC26A3
ITIH2
TFF1
PIP
C2ORF27A
CPA3
OR5E1P
GOLGA8A
GSTA1
SHISA2
KCNK2
SLC28A3
CHI3L1
CPB1
DAPL1
SLC34A2
LCN2
HSD17B2
CLCA4
SLC6A14
EGF
MUC15
XDH
LBTN1A1
ALBA
CSN2
CSN1S1
CSN3
SCGB2A2
SCGB1D2
SCGB2A1
C4ORF7
PROL1
LINC00641
EGFL6
NPR3
TNMD
UCHL1
HSPB6
PLIN4
PDE3B
SLC16A7
THRSP
PRKAR2B
DEFB132
ACVR1C
DGAT2
GPAM
RP1-28O10.1
ADH1A
GYG2
LEP
LPL
PLIN1
ADIPOQ
PCOLCE2
CIDEC
RBP4
SLC19A3
AKR1C2
TIMP4
AQPEP
PCK1
C12ORF39
ME1
TRHDE-AS1
SCD
PLA2G2A
TYRP1
FLG
CLDN1
DSC1
DSG1
LCE2B
SBSN
LCE1B
LOR
KRT2
FLG2
KRTDAP
KRT1
CXCR2
S100A8
PPBP

## Slide 4
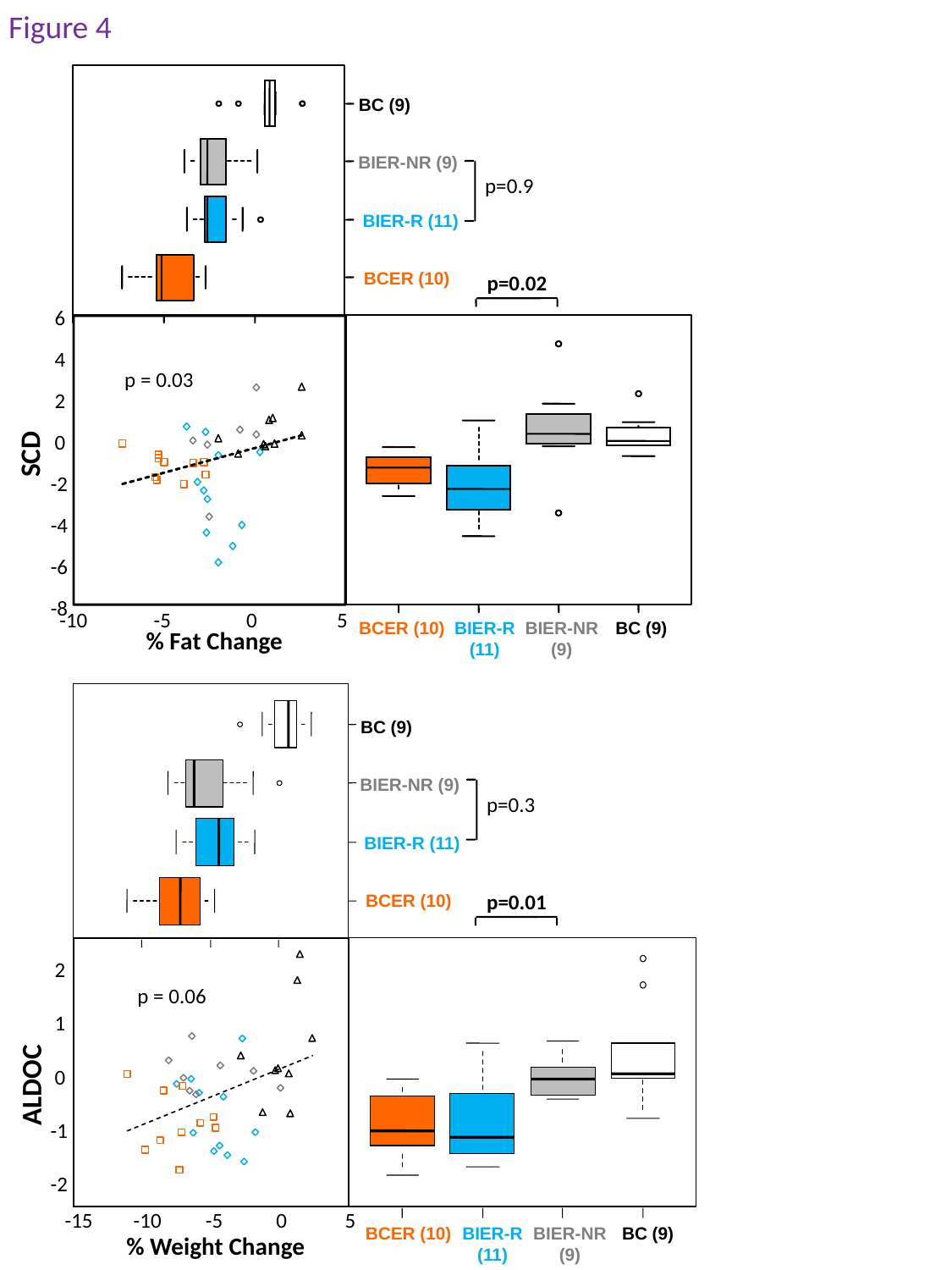

Figure 4
BC (9)
BIER-NR (9)
p=0.9
BIER-R (11)
p=0.02
BCER (10)
6
4
p = 0.03
2
0
SCD
-2
-4
-6
-8
-10
-5
0
5
BCER (10)
BC (9)
BIER-R
(11)
BIER-NR
(9)
% Fat Change
BC (9)
BIER-NR (9)
p=0.3
BIER-R (11)
p=0.01
BCER (10)
2
p = 0.06
1
ALDOC
0
-1
-2
-15
-10
-5
0
5
BCER (10)
BC (9)
BIER-R
(11)
BIER-NR
(9)
% Weight Change

## Slide 5
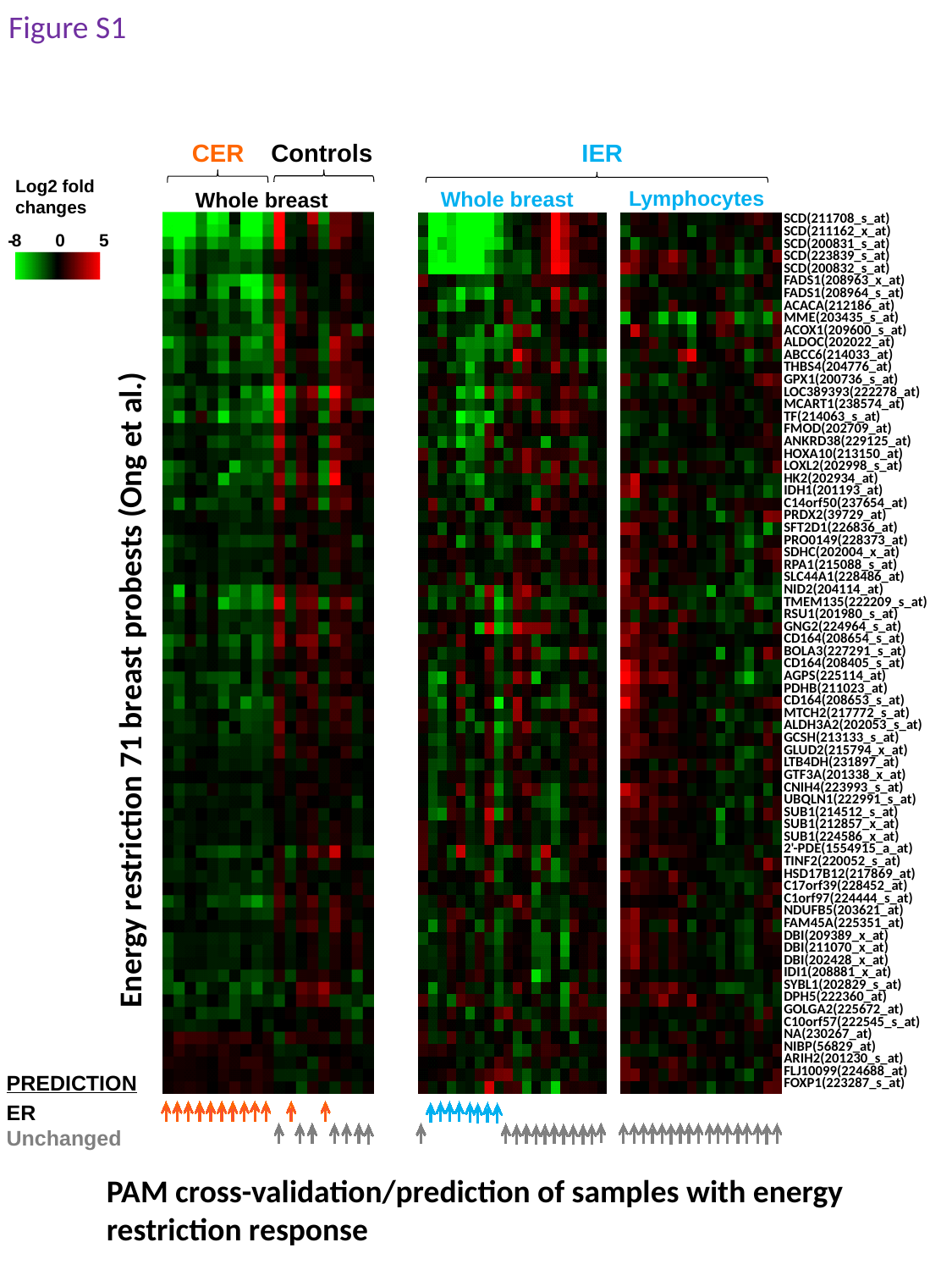

Figure S1
IER
Controls
CER
Log2 fold changes
Lymphocytes
Whole breast
Whole breast
SCD(211708_s_at)
SCD(211162_x_at)
SCD(200831_s_at)
SCD(223839_s_at)
SCD(200832_s_at)
FADS1(208963_x_at)
FADS1(208964_s_at)
ACACA(212186_at)
MME(203435_s_at)
ACOX1(209600_s_at)
ALDOC(202022_at)
ABCC6(214033_at)
THBS4(204776_at)
GPX1(200736_s_at)
LOC389393(222278_at)
MCART1(238574_at)
TF(214063_s_at)
FMOD(202709_at)
ANKRD38(229125_at)
HOXA10(213150_at)
LOXL2(202998_s_at)
HK2(202934_at)
IDH1(201193_at)
C14orf50(237654_at)
PRDX2(39729_at)
SFT2D1(226836_at)
PRO0149(228373_at)
SDHC(202004_x_at)
RPA1(215088_s_at)
SLC44A1(228486_at)
NID2(204114_at)
TMEM135(222209_s_at)
RSU1(201980_s_at)
GNG2(224964_s_at)
CD164(208654_s_at)
BOLA3(227291_s_at)
CD164(208405_s_at)
AGPS(225114_at)
PDHB(211023_at)
CD164(208653_s_at)
MTCH2(217772_s_at)
ALDH3A2(202053_s_at)
GCSH(213133_s_at)
GLUD2(215794_x_at)
LTB4DH(231897_at)
GTF3A(201338_x_at)
CNIH4(223993_s_at)
UBQLN1(222991_s_at)
SUB1(214512_s_at)
SUB1(212857_x_at)
SUB1(224586_x_at)
2'-PDE(1554915_a_at)
TINF2(220052_s_at)
HSD17B12(217869_at)
C17orf39(228452_at)
C1orf97(224444_s_at)
NDUFB5(203621_at)
FAM45A(225351_at)
DBI(209389_x_at)
DBI(211070_x_at)
DBI(202428_x_at)
IDI1(208881_x_at)
SYBL1(202829_s_at)
DPH5(222360_at)
GOLGA2(225672_at)
C10orf57(222545_s_at)
NA(230267_at)
NIBP(56829_at)
ARIH2(201230_s_at)
FLJ10099(224688_at)
FOXP1(223287_s_at)
-
8 0 5
Energy restriction 71 breast probests (Ong et al.)
PREDICTION
ER
Unchanged
PAM cross-validation/prediction of samples with energy restriction response
